# Decision-support tools to build climate resilience against emerging infectious diseases in Europe and beyond

**DOI:** 10.1016/j.lanepe.2023.100701

**Published:** 2023-08-07

**Authors:** Joacim Rocklöv, Jan C. Semenza, Shouro Dasgupta, Elizabeth J.Z. Robinson, Ahmed Abd El Wahed, Tilly Alcayna, Cristina Arnés-Sanz, Meghan Bailey, Till Bärnighausen, Frederic Bartumeus, Carme Borrell, Laurens M. Bouwer, Pierre-Antoine Bretonnière, Aditi Bunker, Chloe Chavardes, Kim R. van Daalen, João Encarnação, Nube González-Reviriego, Junwen Guo, Katie Johnson, Marion P.G. Koopmans, María Máñez Costa, Antonios Michaelakis, Tomás Montalvo, Anna Omazic, John R.B. Palmer, Raman Preet, Marina Romanello, Mohammad Shafiul Alam, Reina S. Sikkema, Marta Terrado, Marina Treskova, Diana Urquiza, IDAlert Consortium, Joacim Rocklöv, Junwen Guo, Jan C. Semenza, Raman Preet, Henrik Sjodin, Zia Farooq, Maquines Sewe, Marina Romanello, Frances MacGuire, Antonios Michaelakis, Evangelia Zavitsanou, Panos Milonas, Dimitrios Papachristos, Marina Bisia, Georgios Balatsos, Spyros Antonatos, Jaime Martinez-Urtaza, Joaquin Triñanes, João Encarnação, Mark Williams, John R.B. Palmer, Berj Dekramanjian, Karl Broome, Otis Johnson, Laurens Bouwer, Maria Máñez Costa, Adriana Martin, Lola Kotova, Thea Wübbelmann, Aditi Bunker, Till Bärnighausen, Fiona Walsh, Marina Treskova, Pascale Stiles, Jerome Baron, Charles Hatfield, Julian Heidecke, Pratik Singh, Shouro Dasgupta, Katie Johnson, Francesco Bosello, Elizabeth J.Z. Robinson, Sara Mehryar, Tilly Alcayna, Meghan Bailey, Marion P.G. Koopmans, Reina S. Sikkema, Pauline de Best, Tijmen Hartung, Ayat Abourashed, Frederic Bartumeus, Jesus Bellver, Catuxa Cerecedo, Rachel Lowe, Martín Lotto Bautista, Bruno Moreira de Carvalho, Chloe Fletcher, Nube González-Reviriego, Marta Terrado, Diana Urquiza, Pierre-Antoine Bretonnière, Julieta Rosenbluth, Marina Corradini, Jaume Ramon, Kim R. van Daalen, Liam Patrick Brodie, Alba Llabres, Ahmed Abd El Wahed, Arianna Ceruti, Uwe Truyen, Chloe Chavardes, Sasha Rodrigues, Anna Omazic, Erik Ågren, Giulio Grandi, Stefan Widgren, Masud Parvage, Martin Bergström, Mohammad Shafiul Alam, Rashidul Haque, Wasif Ali Khan, Tomás Montalvo, Andrea Valsecchi, Laura Barahona, Elisenda Realp, Carme Borrell, Stephan de Roode, Rachel Lowe

**Affiliations:** a Heidelberg Institute of Global Health (HIGH) & Interdisciplinary Centre for Scientific Computing (IWR), Heidelberg University, Heidelberg, Germany; b Department of Public Health and Clinical Medicine, Umeå University, Umeå, Sweden; c Centro Euro-Mediterraneo sui Cambiamenti Climatici (CMCC), Venice, Italy; d Graham Research Institute on Climate Change and the Environment, London School of Economics and Political Science (LSE), London, United Kingdom; e Faculty of Veterinary Medicine, Institute of Animal Hygiene and Veterinary Public Health, Leipzig University, Leipzig, Germany; f Red Cross Red Crescent Centre on Climate Change and Disaster Preparedness, The Hague, the Netherlands; g Centre on Climate Change & Planetary Health, London School of Hygiene & Tropical Medicine (LSHTM), London, United Kingdom; h Centre for Mathematical Modelling of Infectious Diseases, London School of Hygiene & Tropical Medicine (LSHTM), London, United Kingdom; i Health in Humanitarian Crises Centre, London School of Hygiene & Tropical Medicine (LSHTM), London, United Kingdom; j Heidelberg Institute of Global Health, Heidelberg University Hospital, Heidelberg University, Heidelberg, Germany; k Department of Global Health and Population, Harvard T.H. Chan School of Public Health, Boston, MA, USA; l Theoretical and Computational Ecology Group, Centre d’Estudis Avançats de Blanes (CEAB-CSIC), Blanes, Spain; m Institució Catalana de Recerca i Estudis Avançats (ICREA), Barcelona, Spain; n Centre de Recerca Ecològica i Aplicacions Forestals (CREAF), Barcelona, Spain; o Pest Surveillance and Control, Agència de Salut Pública de Barcelona (ASPB), Barcelona, Spain; p Biomedical Research Center Network for Epidemiology and Public Health (CIBERESP), Barcelona, Spain; q Climate Service Center Germany (GERICS), Helmholtz-Zentrum Hereon, Hamburg, Germany; r Barcelona Supercomputing Center (BSC), Barcelona, Spain; s Center for Climate, Health and the Global Environment, Harvard T.H. Chan School of Public Health, Boston, MA, USA; t Three O'clock, Paris, France; u British Heart Foundation Cardiovascular Epidemiology Unit, Department of Public Health and Primary Care, University of Cambridge, Cambridge, United Kingdom; v Heart and Lung Research Institute, University of Cambridge, Cambridge, United Kingdom; w Irideon, Barcelona, Spain; x Department of Viroscience, Erasmus Medical Center, University Medical Center, Rotterdam, the Netherlands; y Laboratory of Insects & Parasites of Medical Importance, Benaki Phytopathological Institute (BPI), Attica, Greece; z Agència de Salut Pública de Barcelona (ASPB), Barcelona, Spain; aa CIBER Epidemiología y Salud Pública (CIBERESP), Madrid, Spain; ab Institut d'Investigació Biomèdica Sant Pau (IIB SANT PAU), Barcelona, Spain; ac Department of Chemistry, Environment, and Feed Hygiene, National Veterinary Institute (SVA), Uppsala, Sweden; ad Department of Political and Social Sciences, Universitat Pompeu Fabra (UPF), Barcelona, Spain; ae Institute for Global Health, University College London (UCL), London, United Kingdom; af Infectious Disease Division, International Centre for Diarrhoeal Disease Research, Bangladesh (icddr,b), Dhaka, Bangladesh

**Keywords:** Climate change, Infectious disease, One Health, Planetary health, Human health, Climate policy, Co-production, Adaptation, Mitigation

## Abstract

Climate change is one of several drivers of recurrent outbreaks and geographical range expansion of infectious diseases in Europe. We propose a framework for the co-production of policy-relevant indicators and decision-support tools that track past, present, and future climate-induced disease risks across hazard, exposure, and vulnerability domains at the animal, human, and environmental interface. This entails the co-development of early warning and response systems and tools to assess the costs and benefits of climate change adaptation and mitigation measures across sectors, to increase health system resilience at regional and local levels and reveal novel policy entry points and opportunities. Our approach involves multi-level engagement, innovative methodologies, and novel data streams. We take advantage of intelligence generated locally and empirically to quantify effects in areas experiencing rapid urban transformation and heterogeneous climate-induced disease threats. Our goal is to reduce the knowledge-to-action gap by developing an integrated One Health—Climate Risk framework.

## Introduction

The emergence, transmission, and geographic range expansion of infectious diseases are driven by global environmental change (including population mobility) and socio-political factors.^1^ Moreover, changes in climate and land use will offer new prospects for viral sharing between wildlife, that were previously geographically separated.^2^ Under these novel circumstances, species redistribution can foster the emergence of zoonotic spillover events that are a potential threat to public health. Of particular concern are zoonoses that rely on invertebrate vectors for their transmission to vertebrate hosts (pherozoonoses). They include insect-borne diseases such as West Nile fever, Dengue, Chikungunya, Leishmaniasis and tick-borne diseases such as tick-borne encephalitis, Lyme disease, and Crimean-Congo haemorrhagic fever. Other zoonoses are also of concern, such as leptospirosis, campylobacteriosis, *vibrio* spp infections or *Escherichia coli* O157:H7, that manifest themselves clinically in humans but only subclinically in animals (cryptozoonoses). What they have in common, is a transmission pathway through an environmental compartment which makes them climate-sensitive. For example, invertebrate vectors are subjected to climate-dependent survival and reproduction rates which affects their transmission potential.^3^ In contrast, zoonoses with a self-sustaining human-to-human transmission cycle, such as SARS-CoV2 and influenza viruses, are less climate sensitive.^4^ The climate-sensitive nature of these zoonoses has been documented for West Nile virus in North America and Europe, which is sustained by wild bird interactions with *Culex* bridge vectors^5^^,^^6^; for chikungunya in Asia, Latin America, North America and Europe^7^; and for dengue globally through *Aedes* mosquitoes^3^; for Lyme disease in North America and Europe, spread by tick vectors.^8^ Whilst there has been a global reduction in malaria incidence in recent decades due to socioeconomic development and healthcare improvements, a warming climate has been associated with the geographical expansion of malaria to higher altitudes and latitude, and smaller outbreaks have re-emerged also in Europe.^9^ Climate change also intensifies the hydrologic cycle, leading to more intense extreme rainfall events, flooding, storm surges and droughts—with implications for water-borne diseases. For example, flooding has been associated with *Leptospirosis* outbreaks,^10^ and warming oceans can accelerate the replication of marine bacteria, such as pathogenic *Vibrio* spp.^11^ Moreover, other highly seasonal water and food-borne diseases—such as *Salmonella* and *Campylobacter*—are strongly associated with air temperature changes during the summer season.^12^^,^^13^

In the context of climate change, the Intergovernmental Panel on Climate Change (IPCC) defines risk as a dynamic interaction between a climate-related hazard, exposure to and vulnerability of affected human or ecological systems.^14^ Hazards, exposures, and vulnerabilities vary across human populations and are subject to uncertainty in terms of magnitude and likelihood of occurrence, depending on spatio-temporal variation in socioeconomic characteristics, and differences in risk management, adaptive capacity, and mitigation strategies. When considering climate-sensitive infectious disease transmission risk, these risk determinants (i.e., hazard, exposure, vulnerability) interact further across domains of human, animal and environmental health. For example, various weather and climatic conditions may influence the reproduction and survival of animals, disease vectors and pathogens. This affects the geographic range, subsequent exposure, and transmission potential of pathogens whilst other biological, ecological, demographic, social, and structural factors may influence vulnerability to these infectious diseases. In that sense, the social determinants of health are important to take into account because they are key to understanding the distribution of infectious diseases and their related factors.^15^ Therefore, indicators that monitor climate-sensitive infectious diseases should account for these complex interactions and their respective contribution to the risk of infectious disease geographical emergence, transmission and spread. To meet these evolving challenges in the preparedness and response to climate-sensitive infectious diseases, a paradigm shift is required that addresses animal, human, and environmental health in an integrated, unifying approach (i.e., using the One Health perspective),^16^^,^^17^ as opposed to siloed approaches (i.e., ones that are focused solely on human health, or on climate risk).

The resilience of the public health to climate-sensitive infectious diseases pertains to its capacity to withstand and effectively manage the threats posed by changes in risk of infectious disease due to a changing climate. It involves the system’s preparedness, strengthening of core capacities and adaptability to new and unexpected stress. Decision-support tools and evidence-based interventions are pivotal in adapting to the new situations by facilitating strategies, resource allocation and implementation of adaptation measures. This includes using decision-support tools for response actions and the ability to optimally scale up interventions under resource constraints. Whilst some tools for short- and long-term prediction of the risk of climate-sensitive infectious disease outbreaks exist, there are still a limited number of operational, usable, and accessible decision-support tools for early warning of possible outbreaks.^18^ Further, much of the data required to support and parameterise these tools is not available, and analyses are currently insufficient.

**Fig. 1:**
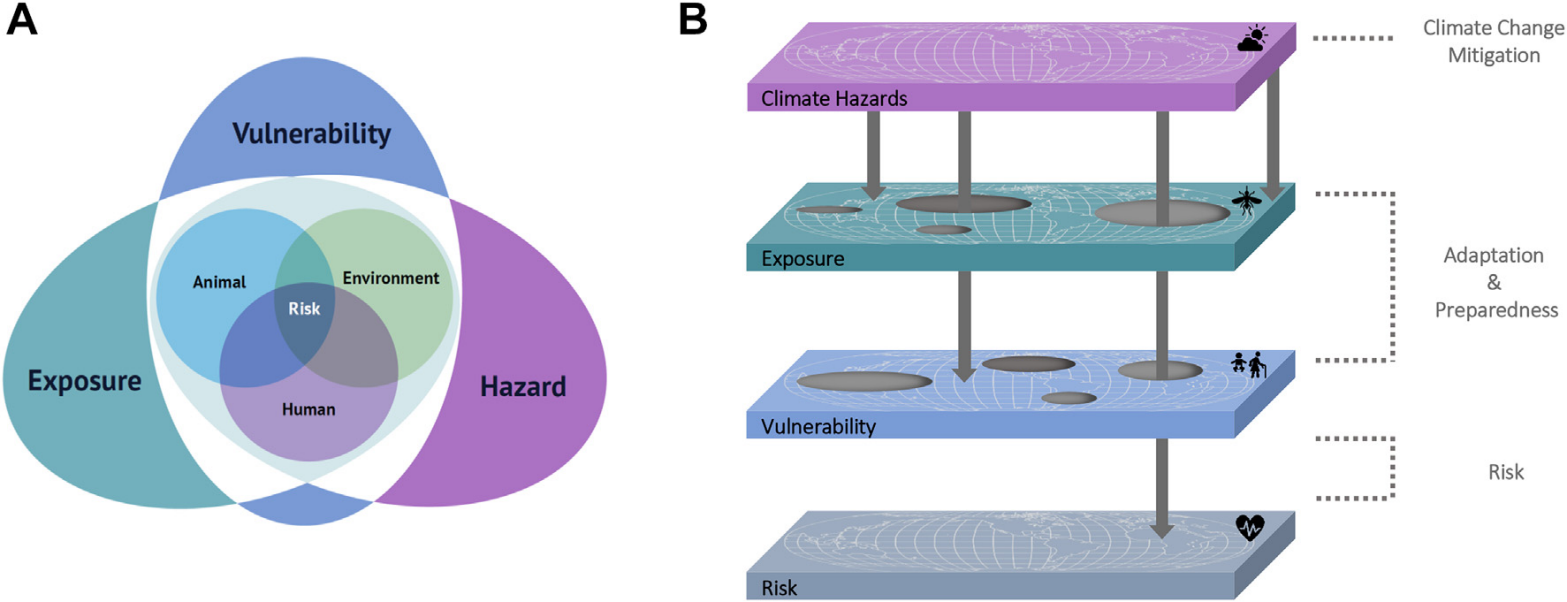
**Integrated One Health–Climate Risk approach.** A.) Illustration of the integration of One Health (i.e., an integrated, unifying approach to animal, human and environmental health) in the IPCC’s framework of risk in terms of hazard (i.e., the occurrence of a climate-related event), exposure (i.e., presence of people, livelihoods, species, ecosystems, resources and infrastructure that can be adversely affected by the hazard), and vulnerability (i.e.,propensity to be adversely affected) to climate change. B.) Nexus of climate hazard, exposure and vulnerability and their associated health risks. Gaps in climate change adaptation and preparedness result in exposure and vulnerability to climate-associated hazards. These gaps constitute a health risk, if they align in time and space. Climate change mitigation can reduce climate hazards, while adaptation and preparedness interventions can reduce exposure and vulnerability to these hazards.

Here, we share our combined experience as the IDAlert consortium which was brought together to research and tackle the geographical emergence and transmission of climate-sensitive infectious diseases in Europe (and beyond), informing cross-sectoral policy while improving the long-term and upstream climate resilience of health systems to infectious disease risks. Building on both the IPCC’s framework to climate risk and the One Health perspective to integrated animal, human and environmental health surveillance, we propose a holistic and comprehensive approach to tackling the emergence, transmission, and dispersion of infectious diseases (Fig. 1). We do it by applying a transdisciplinary “Integrated Knowledge-to-Action framework” which combines applied research streams to co-produce evidence-based decision-support tools and solutions in collaboration with a diverse set of policy stakeholders. Additionally, we incorporate routinely collected and novel data streams and consider social vulnerabilities to advance innovative and fair solutions for climate resilience.

## Search strategy and selection criteria

We identified references for this Health Policy paper searching the PubMed database and selecting based on the article's relevance to the scope of this research. The objective was to identify peer-reviewed articles, published in English between January 2012 and December 2022, that focused on predicting the risk of climate-sensitive infectious diseases in Europe and exploring control strategies. The search strategy encompassed the concepts of "climate change", "climate-sensitive infectious diseases", and "Europe". For the concept of climate change, the search terms used included "climate change", "climate variability", "global warming", and "climate change adaptation". The concept of climate-sensitive infectious diseases involved search terms such as "dengue", "lyme disease", "malaria", "tick-borne encephalitis", "chikungunya", "West Nile", "Crimean-Congo haemorrhagic fever", "leptospirosis", "campylobacteriosis", "salmonellosis", "vibrio", "*Escherichia coli*", "zoonotic", "insectborne", "vector-borne", and "water-borne", and relevant synonyms. The selection of articles for inclusion in this study was guided by expert judgment of the articles' originality and relevance to the broad scope of the paper. The focus was primarily on articles investigating early warning systems, decision support tools, co-benefits of climate adaptation strategies, and epidemic intelligence. In total, 70 articles were selected as the evidence basis for this paper.

## Integrated knowledge-to-action framework

**Fig. 2:**
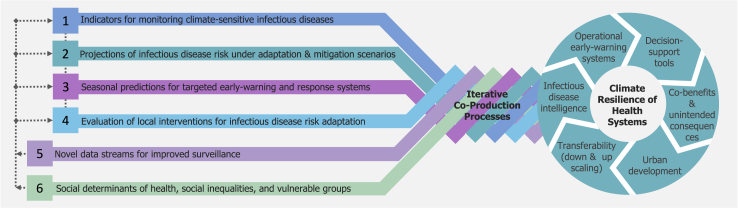
**Integrated Knowledge-to-Action framework is a proposed approach for generation and transfer of knowledge and research activities on hazards, exposures, vulnerabilities, and risk while integrating environment, animal, and human health to action that creates pathways to a wider societal impact and building-up of health systems’ climate resilience.** The framework integrates four major research streams (1–4) and two approaches to research that cut across and are incorporated in all knowledge and evidence generation activities (5–6). A participatory approach is applied to engage knowledge-users and stakeholders into iterative processes of co-design, co-development, and co-dissemination of research. The wheel illustrates the short- and long-term outputs that lead to societal impacts, improve the climate resilience of health systems, and benefit the society at large.

**Fig. 3:**
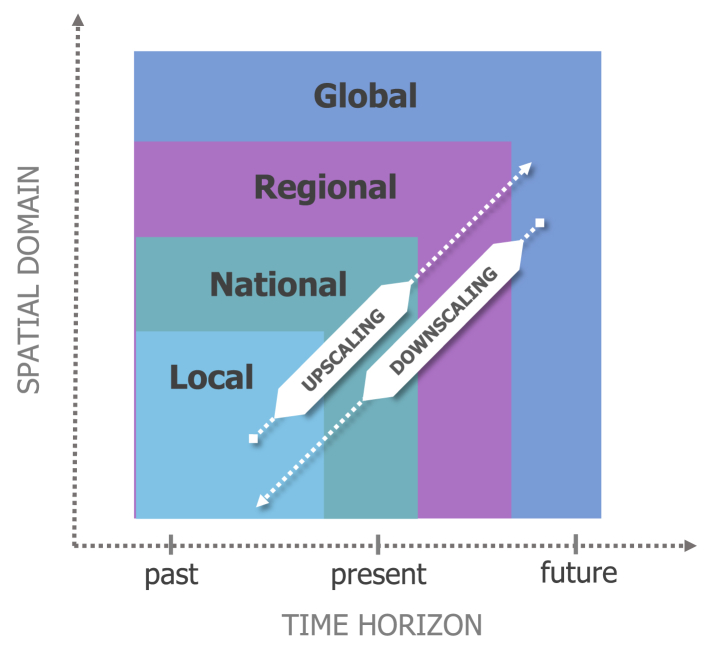
**Spatio-temporal domains at which the integrated One Health–Climate Risk tools are being developed.** Indicators developed using global products can be downscaled using information collected at higher-resolution spatial scales and incorporated into regional or local decision support systems. Meanwhile, locally collected data can inform infectious disease models and improve assessments of disease risk across larger geographic areas (i.e., national, regional, or global). Indicators and models can be formulated and applied to track historical changes (past), predict the probability of emergence and outbreaks from subseasonal to seasonal time scales, and project changing risk patterns in the long-term (e.g., until the end of the century), given different GHG emission pathways, population changes, and levels of adaptation and mitigation.

Our proposed Integrated Knowledge-to-Action framework (Fig. 2) aims to enable the co-production of policy-relevant innovative decision-support tools, research and data streams capturing climate change related hazards, exposure, vulnerabilities and risks surveillance methods, and interventions to improve resilience to climate-sensitive infectious disease risks using a One Health approach. Because the problem cuts across science, economics, and society, methodologically we combine different disciplines building upon the integrated One Health-Climate Risk approach (Fig. 1) and is designed to operate across several spatial domains (local, national, regional, and global) and temporal scales (tracking historical changes, short-term predictions, and long-term projections; Fig. 3). Using this proposed framework, we conceptualise surveillance methods that take advantage of a broad set of information sources. Local case study research and data streams need to be combined with further empirical and epidemiological data, modelling results, and expert assessment to co-develop i) indicators that track historical climate-sensitive infectious disease risk and project future impacts, ii) early warning and targeted response systems, and iii) methods to evaluate (existing) climate adaptation and mitigation interventions in specific study sites.

Our proposed approach strives to increase the quality, effectiveness and uptake of the research outcomes and the development of user-oriented decision-support tools. This is in contrast with more traditional fundamental science approaches where there is little engagement of end-users or research is selectively “delivered” to the potential end-users.^19^^,^^20^ The applied science approach has been demonstrated to more appropriately assess and adapt to inequities relevant to the benefits and drawbacks of climate change policies, and to protect populations–particularly those most vulnerable–from maladaptation and its associated unintended consequences.^21^, ^22^, ^23^ The co-production processes within this framework consist of iterative collective needs assessments, joint prioritisation, inclusive decision-making, participatory research, and shared knowledge generation, with an ongoing process of review and learning.

This approach aims to ensure that relevant stakeholders from various disciplines, sectors, and levels of decision authority are involved throughout the process and that user needs (i.e., affected individuals and communities) are taken into account from the start; a co-design of the research questions that meet collective interests and needs is the first phase of the overall co-production process, and the outcomes are taken forward into the co-development phase.

Addressing research legitimacy and ethics is important for studies at the intersection of science, policy, and community.^24^ This is particularly so for studies such as ours which aim to work with multiple stakeholders, and incorporate citizen science approaches, to develop a deep and nuanced understanding of how relevant policies reach and affect different populations, how those populations choose to, or are able to, engage with these policies, and overall outcomes, specifically whether societal inequalities are reduced or increased. When using qualitative research approaches, we will be especially cognisant of consensus, wide participation, and gathering a range of views. Participatory implementation of the framework is expected to increase the likelihood that research outputs are translated into useful and usable information and knowledge to help inform policy and decisions that result in the reduction of climate change-induced infectious disease risks. The final step in the process is the co-dissemination and exploitation of research outputs, which includes leveraging stakeholder networks to maximise impact, ensuring the relevance of the research for health and climate change decision-makers, and that outputs align with end-user needs.

The proposed framework can be used to identify and address a diverse set of research questions to enhance the co-development and deployment of novel surveillance, monitoring and forecasting systems, predictive models, and early warning systems for climate-sensitive disease threats. Here, we describe the transdisciplinary research streams within our framework.

### Indicators for monitoring climate-sensitive infectious disease

Current monitoring and risk assessments show that parts of Europe are becoming more climatically suitable for infectious disease transmission, driven by environment, vectors, and ecosystem impacts on wildlife in interaction with humans. Within this context, indicators can be used to track and communicate these complex climate-health trends in a more accessible format to inform advocacy, policy-making and science.^25^ For example, the Lancet Countdown on Health and Climate Change in Europe has developed climate-sensitive infectious disease indicators to track changes in the climatic suitability for the transmission of water-borne and mosquito-borne diseases.^26^ However, pathogen incidence in animal reservoirs, or changes in populations and movements of those animals that can lead to spillover risk to humans, have not been addressed. Seasonal bird migrations, for example, have been linked to the spatial spread of avian pathogens with zoonotic potential.^27^, ^28^, ^29^ Although their precise role in disease dynamics is not yet fully understood,^30^ shifts in animals’ migratory behaviour, routes, and timing as a response to changes in climate can be important indicators of disease emergence. Currently, available indicators track non-cholera *Vibrio*, West Nile virus, dengue, chikungunya, Zika, and malaria, which have been operationalised in the European Climate and Health Observatory as interactive visualisations to inform and support decision-making.^31^ These indicators show that changing environmental conditions are shifting the suitability for the transmission of mosquito-borne diseases further north and that the transmission season is lengthening in southern, eastern, and central Europe. This is particularly concerning given that in these European risk regions, the human incoming travellers from areas reporting dengue outbreaks at the global scale are estimated to have increased substantially over the last 30 years and this has been shown highly correlated to the importation of the virus.^26^^,^^32^ The percentage of coastal waters in Europe with suitable conditions for the transmission of pathogenic non-cholera *Vibrio* is also increasing.^33^ However, indicators for a wider range of zoonotic diseases and spillover risk from animals to humans, via vectors and/or reservoirs, such as Lyme disease, tick-borne encephalitis, leishmaniasis and leptospirosis, hantavirus and respiratory viruses are currently missing. Crucially these infectious diseases should be monitored in light of the unifying One Health–Climate Risk approach to capture more accurate estimates of “true” disease risk and burden shared among human and animal populations (Fig. 1).

### Projections of infectious disease risk under adaptation and mitigation scenarios

Short-term and long-term projections of climate-sensitive infectious disease risk can be used to underpin climate mitigation and adaptation policies across sectors in society at different temporal and spatial scales (Fig. 3). For Europe, high-resolution regional climate projections have been developed for a range of plausible pathways and scenarios that include the relationship between human choices (e.g., continued dependence on fossil fuels or deliberate climate action) and greenhouse gas (GHG) emissions, GHG concentrations and levels of warming to predict future change (often up to 2100).^34^ Combining climate-sensitive disease indicators with short-term and long-term climate projections can be useful to illustrate potential impacts of climate change on climate-sensitive infectious disease risks and inform relevant policy development.^9^^,^^35^, ^36^, ^37^, ^38^ However, currently, there is a lack of climate-sensitive infectious diseases projections in Europe.^39^ Notably, despite the importance and climate-sensitivity of zoonotic diseases,^40^ these diseases as well as their animal reservoirs, are particularly under-studied. Moreover, the unintended consequences of adaptation and mitigation policies, e.g., expansion of urban green and blue spaces for reducing heat impacts, flood risk reduction, and biodiversity conservation, may simultaneously increase the risk of some infectious diseases and therefore is critical for consideration in risk assessments.

Our projections will be based on a range of future climate change and socioeconomic scenarios under the Coupled Model Intercomparison Project Phase 6 (CMIP6) for short-term (2030), medium-term (2050), and long-term (2100). Our goal is to provide policy relevant projections under the following scenarios:

1. SSP1-RCP1.9 (Paris target): informing the Paris Agreement target of 1.5 degree C above pre-industrial levels, CO_2_ emissions are cut to net zero around 2050. Global temperature will likely increase by 1.5 °C by 2100.
2. SSP4-RCP3.4 (closest to Glasgow commitments): a gap-filling mitigation scenario which fills in the range of low forcing pathways. Global temperature will likely increase by 2.7 °C by 2100.
3. SSP2-RCP4.5 (middle of the road): CO_2_ emissions follow the current trajectory before starting to fall mid-century but do not reach net-zero by 2100. Socioeconomic factors follow their historic trends with slow progress toward sustainability and development and income growing unevenly. Global temperature will likely increase by 2.95 °C by 2100.
4. SSP3-RCP7.0 (near catastrophic): emissions and temperatures rise steadily and CO_2_ emissions roughly double from current levels by 2100. Countries become more competitive with one another, shifting toward national security, and ensuring their own food supplies. Global temperatures will likely increase by 3.6 °C by 2100.

These projections and indicators will combine robust empirical and epidemiological exposure-response functions estimated for the case study sites (city or regional) and for the European-level. The projections will also include potential effects and impacts of upstream mitigation policies (greenhouse gas reduction, under different SSP and RCP scenarios), as well as adaptation policies developed within and outside the health sector.

### Seasonal predictions for targeted early-warning and response systems

Climate information, including observations, reanalysis and forecasts are key components of early warning systems for climate-sensitive infectious diseases. The goal of a climate-driven early warning system is to provide advance warnings of increased outbreak risk with enough lead time to deploy interventions that can mitigate the impact of an imminent outbreak. This can be done by either incorporating latencies in how weather affects disease outcomes (e.g., the temperature in the previous month is used to predict the number of cases this month) or through the use of climate forecast products as inputs for infectious disease forecasting models. Subseasonal-to-seasonal climate predictions, ranging from several weeks to months ahead, have greatly improved over the last decade and have the potential to improve the timeliness and impact of public health response measures. However, despite improvements, climate predictions are often provided in a format that is difficult to understand, process or apply to public health applications, such as infectious disease epidemiological modelling.^41^ Further research and close collaboration with climate scientists are needed to broaden knowledge on the usability and skill of climate predictions as well as the requirements of vector and disease control and prevention teams. Despite the established use of early warning systems in other fields, such as disaster risk reduction or agriculture,^42^ the integration of climate-informed early warning systems within the health sector remains limited to date. Climate-driven early warning systems prototypes have been developed to predict the probability of exceeding user-defined infectious disease outbreak thresholds, to provide disease control teams with sufficient lead-time to implement timely interventions, such as fogging and educational campaigns.^43^, ^44^, ^45^, ^46^ However, due to a lack of investment and capacity building, prototypes rarely transition into operational and sustainable tools used by health systems and policymakers.

### Evaluation of local interventions for infectious disease risk adaptation

Adaptability is a fundamental attribute of a resilient public health system. The ability to adjust strategies, upscale interventions and allocate resources based on changing conditions is essential for effectively addressing emerging challenges. Evidence-based mitigation and adaptation interventions are pivotal in facilitating this ability to adapt. Rigorous evaluation methods of interventions from the One Health perspective can improve the design of local, sustainable, long-term, upstream solutions and interventions to enhance resilience to climate-sensitive infectious diseases and help identify and monitor unintended consequences, i.e., not anticipated undesirable health outcomes. For example, whilst certain regions in Europe upscale climate change adaptation and mitigation based on decisions in various sectors of society, such as open water to protect from floods, improved storm drains, expansion of urban parks and restoration of wetlands, little is known about the unintended impacts of these control actions in terms of vector-borne zoonotic diseases, and how to control and manage any potential negative consequences Nor is it known how efficacy is mediated by other factors, such as residents’ knowledge and behaviour, weather conditions, landscape characteristics, city-level greening, and water use practices–particularly in the context of a changing climate and expanding disease-vector mosquito ranges. Therefore, it is necessary to quantify the causal impacts of climate adaptation and mitigation interventions on climate-sensitive infectious disease risk using experimental (e.g., randomised control trials) and quasi-experimental approaches (e.g., difference-in-differences estimation (DiD), geographical regression discontinuity design, instrumental variable approach), and leveraging data collected from different sources. Quasi-experimental methods are advantageous in evaluating the causal impact of, for example, landscape or infrastructural interventions (e.g., wetland construction, water storage, storm drain replacement, urban greening, and anti-flood works after a fire) on the risk of vector-borne and zoonotic diseases because of the infeasibility to randomise such interference.^47^^,^^48^ Interventions such as drain replacement, biological larviciding, landscape transformation, flood adaptation, and urban greening vary over space and time (e.g., because of seasonal baseline differences in mosquito density), creating DiD study design opportunities which allow controlling for unobserved time-invariant and time-varying confounding.^49^ In addition to assessing the technical effectiveness of interventions, as part of an overall performance evaluation, it is equally important to consider any unintended consequences of the intervention, particularly on health outcomes, and to determine the economic "value" of the intervention. Neither is simple. But both are crucial for enhancing decision-making processes and providing a more comprehensive understanding of an intervention's net impact. For example, there is potential for urban green-blue solutions to create new breeding sites for infection-carrying vectors. Further, while the costs of an intervention are likely to be relatively easy to determine, the benefits may be diffuse and hard to quantify in monetary terms. By incorporating such evaluations, decision-makers are empowered to make informed choices and adjust strategies to address emerging infectious disease risks effectively.

### Novel data streams for improved surveillance

Cutting across research streams 1–4 are technology and methods for improved surveillance. For vector-borne and zoonotic diseases, novel sources of data are proving to be increasingly useful in augmenting traditional surveillance methods. A number of surveillance systems, based on novel data streams have been operationalized in Europe for public health purposes:

• *Aedes japonicus* was discovered in Spain in 2019 through a Citizen Science approach.^50^ The MosquitoAlert platform was developed to identify invasive mosquitoes by engaging the public to report their findings. The presence of this invasive mosquito species was subsequently verified by VectorNet entomologists at ECDC/EFSA.
• An early warning system (EWS) for the suitability of *vibrio* spp. infections in marine environments globally was developed using remotely sensed environmental and climatic data.^33^ This EWS has been operationalized by ECDC and alerts are sent to state epidemiologists in countries with high environmental and climatic suitability for *vibrio* spp. infections in their coastal waters.
• Big data were used to predict the spread of chikungunya in France and Italy in 2017 from the epicentre of the outbreak to other areas at risk.^7^ Air passenger volume from IATA was used to predict the importation risk of viraemic passengers in areas climatically suitable in Europe. The risk of dispersion was estimated with Twitter data to model the population movement in the area of the outbreak to estimate the dispersion of chikungunya in Europe.
• The operationalization of a rapid point-of-use field detection methods with a suitcase laboratory (Diagnostics-in-a-Suitcase) is a rapid, inexpensive, and simple diagnostic approach to circumvent central laboratories in difficult field settings, for timely public health action.^51^
• Eco-climatic determinants for West Nile virus outbreaks in Europe were defined using machine learning algorithms and tested against outbreak data.^5^ Deviations from average Spring temperature can now be used as an EWS to direct vector control efforts and outreach to the public in order to proactively intercept emerging West Nile virus outbreaks.

Citizen science, for example, offers a highly scalable method for tracking arthropod vectors like mosquitoes and ticks,^52^, ^53^, ^54^, ^55^, ^56^, ^57^ as well as wildlife observations and monitoring. Digital tools can be used to implement health-related surveys (e.g., on knowledge, action, behaviour, and practice of mosquito-bite prevention) and study human mobility patterns in ways that capture information most relevant in the context of climate change. Artificial intelligence is being used to automatically classify mosquitoes and other vectors based not only on photographic image recognition but also on wingbeat characteristics detected in real-time with acoustic or optical sensors integrated into traps and networked through the ‘Internet of things’ traps.^58^^,^^59^ New DNA and RNA sequencing technologies are making it easier to rapidly screen for pathogens and vectors in field settings, particularly through the use of mobile suitcase labs.^51^ Moreover, applying such methods to environmental sample types, rather than human and animal samples can save time and reduce costs, and avoid human and animal discomfort.

To produce near-real-time open data connected to expert validation platforms that can be shared with local stakeholders for decision-making and intervention, there is a need to build on and improve participatory and crowd-based strategies for disease vector data collection. This can entail both technological improvements of currently available tools and the maintenance of active communities of scientific experts, citizen scientists and end users–for example, building on the Mosquito Alert citizen science system,^53^ and the wildlife disease surveillance programs in Sweden and the Netherlands.^60^, ^61^, ^62^, ^63^ Further, automated surveillance of vector activity can be done by networked and automated smart traps with mosquito differentiation capabilities. This new surveillance technique allows for counting that can provide automatized cost-efficient time series data, thus providing both abundance and dynamics of gravid female activity.^64^ Algorithms are being trained using both expert- and crowd-annotated image datasets to improve the scalability of citizen-science data validation. Combining bioacoustic recordings of live insects in the lab and field data acquired by smart-trap networks in the study sites, the machine learning algorithms for IoT mosquito sensors can be updated, rendering these more precise and effective. Furthermore, targeted mobile suitcase labs can be deployed to screen a variety of vectors, hosts, and reservoirs for pathogens and to identify host species in vector blood meals whilst avoiding the problems of sample degradation through handling, storage and transportation associated with traditional molecular biology approaches. Advances in sample types and matrices used can also increase possibilities for citizen science as well as increase sample sizes. Examples are the use of feathers for bird surveillance,^65^ wastewater for human pathogens,^66^^,^^67^ or slaughterhouse monitoring.^68^ Human mobility and activity-space data can be collected using active mobile phone positioning linked to digital knowledge, attitudes and practices (KAP) surveys and socioeconomic population data. These novel data streams in combination with already available surveillance and register data can be used to investigate determinants of and inequalities in exposure to infectious diseases and their vectors across population strata (e.g., gender, ethnicity, migration status, occupation, income). In summary, these novel data streams lend themselves to connect the surveillance efforts in human, animal, and environmental health towards a continuum of surveillance across these disciplines.^11^

### Social determinants of health, social inequalities, and vulnerable groups

The consideration of social vulnerabilities is further a cross-cutting research stream. This is motivated because similar to other climate-related health impacts, disadvantaged groups defined by axes of inequality such as socioeconomic status, gender, geographical area, etc., suffer disproportionately from infectious diseases.^69^^,^^70^ Moreover, the intersectionality of these axes has to be acknowledged because it can increase the incidence of such diseases. With climate change expected to further exacerbate existing social inequalities, the impacts on the most vulnerable groups are expected to worsen. Evidence on COVID-19-related impacts showed the differentiated effects of an emerging pandemic on different population groups,^71^ effects that changed through the waves, with the pandemic being referred to as “*the great unequalizer*”. Therefore, it is important that the work in this area includes a strong emphasis on social justice, giving special attention to correcting patterns of unequal responsibilities and harms (including infectious diseases) of climate change.^72^

Preparedness and response to climate-sensitive infectious disease should include explicit attention to identifying vulnerable populations and targeting the social determinants of health such as the structural inequalities resulting from the interface of populations with socioeconomic, political and cultural or normative hierarchies.^73^ Monitoring the emergence, transmission, and spread of climate-sensitive infectious diseases, combined with a detailed understanding of where the most vulnerable and marginalised populations are located and how they can best be reached, can help to reduce impacts on high-risk, and hard-to-reach population groups. Therefore, within our proposed research framework we aim to identify sub-population groups (in terms of age, gender, income, socioeconomic position, occupational settings, migration status, etc.) most at risk from climate-sensitive infectious diseases by integrating multiple survey datasets (e.g., EU Statistics on Income and Living Conditions and EU Labour Force Survey), health registry data, and One Health surveillance data. Using this transdisciplinary approach, inequality and local context can be explicitly incorporated to co-produce stratified impacts of climate change-induced infectious diseases on society and contribute to effective policy changes.

In terms of economic (costs and) benefits, one of the components in our framework will investigate the higher order costs of infectious diseases and the benefits of adaptation and mitigation measures, and estimate economic costs. The aim is to quantify how infectious diseases-related mortality and morbidity impacting on the labour force supply/productivity and healthcare expenditure can affect EU countries’ GDP, sectoral economic activity, competitiveness, and stress public budgets. As such the framework accounts for direct and indirect economic effects in cost-benefit analysis from infectious disease response and prevention with climate policy and climate change impacts. Future strategies for mitigation and adaptation linked to cost-benefit assessment and society impacts will be incorporated in the projections of the long-term economic benefits and costs of health interventions and adaptation in the health sector under different climate change scenarios.

## Policy context

Science is instrumental in developing and advancing novel approaches for climate resilience and the One Health perspective has been identified by the Quadripartite Collaboration (FAO/UNEP/WHO/WOAH) as the main way forward.^74^ However, implementation and uptake of these approaches depend on their desirability, viability, and feasibility from the decision- and policy-making perspectives. The described interdisciplinary and co-production approach is designed to contribute to more effective and just policy-making that has a wide reach and positive benefits across all sectors of society, particularly for the most vulnerable, including for example lower-income households and others in disadvantaged socioeconomic positions, people on the move, and those facing gender discrimination. Local insights and monitoring guide pan-European level decisions through upscaling and engaging with stakeholders. Based on this, evidence-based decision-making can be informed by indicator trends, early warning tools and platforms, and citizen science for a better understanding of local infectious disease risks and a mapping of vulnerable populations. The proposed approach can also provide an understanding of the impacts and cost-benefits across various mitigation and adaptation efforts.

This approach is particularly timely in a post-pandemic world where a zoonotic disease just created a major health security threat and given the ambition of the Quadripartite to increase the uptake and influence of One Health perspectives at various levels in society and by embracing the increased need to prevent and prepare for emerging infectious diseases, which relates directly and indirectly to climate change mitigation and adaptation policies. The Quadripartite also emphasise the development and strengthening of the scientific knowledge exchange and evidence creation, in which the transdisciplinary approach we propose is of key importance. The approach can at the same time play an essential role in the EU’s ambition to improve the knowledge base for climate and health adaptation, whilst championing the European Green Deal,^75^ EU4Health,^76^ and the EU Adaptation Strategy.^77^ At present, policies and policy discussions have tended not to take into account the impacts of climate adaptation and mitigation-driven transformations on infectious disease outcomes. As such, some dimensions of health outcomes could be worsened rather than benefit from such policies such as the unintended creation of more mosquito breeding sites through climate policies focused on some nature-based solutions in highly populated areas. Climate change and One Health data need to be further integrated to assess the development and uptake of climate policy as it relates to the emergence, transmission and spread of infectious pathogens to highlight how preparedness in the EU unfolds with policy implementation. Further, engaging critical stakeholders in the co-creation and co-design of research from the start is an important step in ensuring long-lasting impacts on EU climate policy, and providing new evidence and tools for the European Green Deal to strengthen population health and societal resilience to climate change and increase chances of knowledge to action transfer.

## Conclusions

Our ambitious research approach involves monitoring, predicting, testing, evaluating and upscaling innovative surveillance and citizen science to increase preparedness and response to the growing threat of climate-sensitive infectious disease; estimating the effectiveness of interventions and adaptation strategies; and an assessment of the unintended consequences of climate adaptation and mitigation policy on climate-sensitive infectious diseases. We will test the approach in a number of case studies focusing on different bio-geographical, socioeconomic and political particularities. The multidisciplinary research and co-production are designed to better inform inter-sectoral policy-making, and guide public health authorities, animal health and environmental services including climate risk management. Altogether we hope it will enhance evidence to safeguard the health of the populations in Europe and beyond from the transmission and geographical emergence of infectious pathogens in the context of rapid environmental and climate change.

## Contributors

Joacim Rocklöv, Rachel Lowe, Jan C. Semenza, Shouro Dasgupta, Elizabeth J. Z. Robinson, Marina Treskova, Cristina Arnés-Sanz and Kim R. van Daalen prepared the first draft of the manuscript. All the authors contributed to the conceptualisation, and critically reviewed and edited the manuscript.

## Declaration of interests

All authors declare no competing interests.
